# Ovarian Myxoma in a Patient With Missed Abortion: A Diagnostic Dilemma

**DOI:** 10.7759/cureus.118225

**Published:** 2026-10-20

**Authors:** Reenu Kanwar, Shubham Bidhuri, Madhumita Patel, Manjula Sharma

**Affiliations:** 1 Obstetrics and Gynaecology, Vardhman Mahavir Medical College & Safdarjung Hospital, New Delhi, IND

**Keywords:** adnexal mass, immunohistochemistry, mature cystic teratoma, mesenchymal tumor, missed abortion, ovarian cystectomy, ovarian myxoma, scrape cytology

## Abstract

Ovarian myxoma is a rare benign mesenchymal tumor. It can be completely asymptomatic and is difficult to differentiate from other ovarian masses before surgery and final histopathology. We report a case of a 28-year-old primigravida who presented with a missed abortion at 11 weeks. Transvaginal ultrasonography incidentally showed a 6.5 × 5.7 cm complex solid-cystic right adnexal lesion in the pouch of Douglas. She was asymptomatic from the mass. After dilatation and evacuation for missed abortion, the patient was evaluated for an ovarian mass, and tumor markers were found negative. Repeat ultrasonography showed a heterogeneous lesion with coarse hyperechoic foci and posterior acoustic shadowing, suggesting a right ovarian teratoma. The patient was taken for laparotomy, wherein an 8 × 8 cm solid-cystic mass was found in the right ovary, and right ovarian cystectomy was done. Intraoperative scrape cytology came back negative for malignancy and showed columnar cells, squamous cells, and mature adipose tissue, which favored a benign cystic teratoma. Histopathology showed a thick collagenous capsule and stellate cells in abundant myxoid stroma without atypia. On immunohistochemistry, the tumor cells were positive for vimentin and negative for desmin and cytokeratin. The final diagnosis was ovarian myxoma. The postoperative course was uneventful, and the patient was discharged on postoperative day 5. There was no recurrence at one-year follow-up. This case shows that ovarian myxoma can mimic a mature cystic teratoma on imaging, gross examination, and cytology, and that the final diagnosis depends on histopathological examination (HPE) supported by immunohistochemistry.

## Introduction

Ovarian myxoma is a rare benign mesenchymal tumor. It is mostly seen in women of the reproductive age group [1]. On histopathological examination (HPE), it consists of bland spindle or stellate cells lying in abundant myxoid stroma. It usually presents as a unilateral ovarian mass [1]. It can be asymptomatic and is often detected as an incidental finding. Due to its rarity and nonspecific appearance on imaging, ovarian myxoma is difficult to diagnose before surgery [1,2]. The differential diagnosis includes mature cystic teratoma, ovarian fibroma or thecoma with myxoid change, massive ovarian edema, and other myxoid ovarian tumors. Therefore, HPE supported by immunohistochemistry is essential for a definite diagnosis [1,2]. We report a case of ovarian myxoma found incidentally during the evaluation of a missed abortion in early pregnancy. On imaging, on gross examination, and on intraoperative scrape cytology, the lesion was thought to be a mature cystic teratoma. This case highlights the diagnostic challenge of this rare tumor and the importance of histopathology in confirming the diagnosis.

## Case presentation

Clinical presentation and imaging

A 28-year-old primigravida presented at 11 weeks with bleeding per vaginum. She had no significant previous medical or surgical history and reported previous normal menstrual cycles. Transvaginal ultrasonography confirmed a single intrauterine fetus with no cardiac activity. An incidental 6.5 × 5.7 cm complex solid-cystic lesion was also seen in the pouch of Douglas. She had no symptoms from the mass. Tumor markers (CA-125, CEA, CA19-9, AFP, and HCG) were sent and were negative. She underwent dilatation and evacuation for the missed abortion and was discharged with advice to follow up for evaluation of the mass. On follow-up after four weeks, the radiology report documented a complex right adnexal lesion measuring 6 × 4.8 × 6.5 cm, reported as heterogeneous and hypoechoic with coarse hyperechoic foci causing posterior acoustic shadowing and a few cystic spaces. The right ovary could not be seen separately. The left ovary looked normal. The uterus was anteverted, and the endometrial thickness was 8 mm. There was no free fluid. An impression of right ovarian teratoma was made. The images from this study were not available for reproduction.

Surgery

After consent and preanesthetic evaluation, she was planned for laparotomy. Intraoperatively, an 8 × 8 cm solid-cystic mass was found in the right ovary, compressing healthy ovarian tissue. The left ovary was bulky and had a suspicious 2 × 2 cm solid area with a small surface protrusion. Right ovarian cystectomy was done, and the specimen was sent for intraoperative scrape cytology. It was negative for malignancy and showed columnar cells, squamous cells, and fragments of mature adipose tissue, with a few areas of chronic inflammation. A provisional diagnosis of benign cystic teratoma was made. Biopsies of the suspicious area of the left ovary and of the omentum were also taken.

Gross and microscopic findings

The specimen was a solid-cystic tumor measuring 6 × 6 cm, with a glistening capsule and a breach in the capsule (Figure 1). The outer surface was pearly white and smooth, with a 1.5 × 1 × 0.8 cm protrusion at one site. On cut section, the tumor was solid-cystic. The cyst was 2 cm in diameter, and the solid areas were grayish yellow (Figure 2). No hair, bone, or tooth was seen.

**Figure 1:**
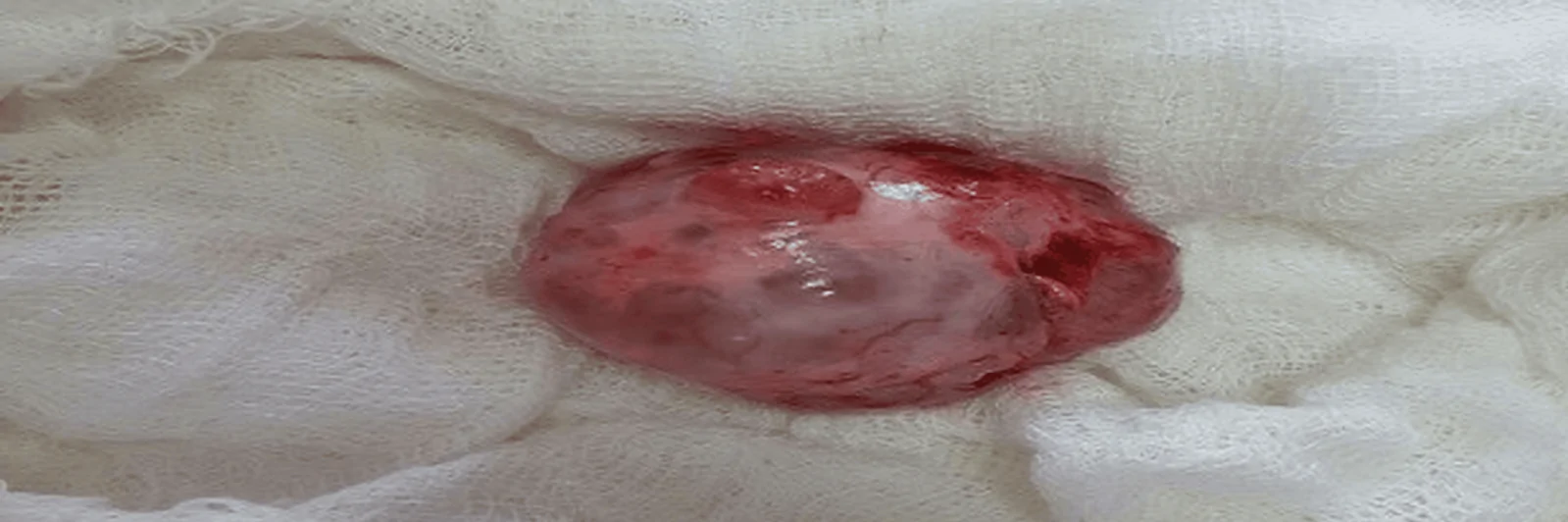
Gross appearance of the ovarian mass Gross specimen of the right ovarian mass, measuring 6 x 6 cm with a glistening capsule and focal capsular breach

**Figure 2:**
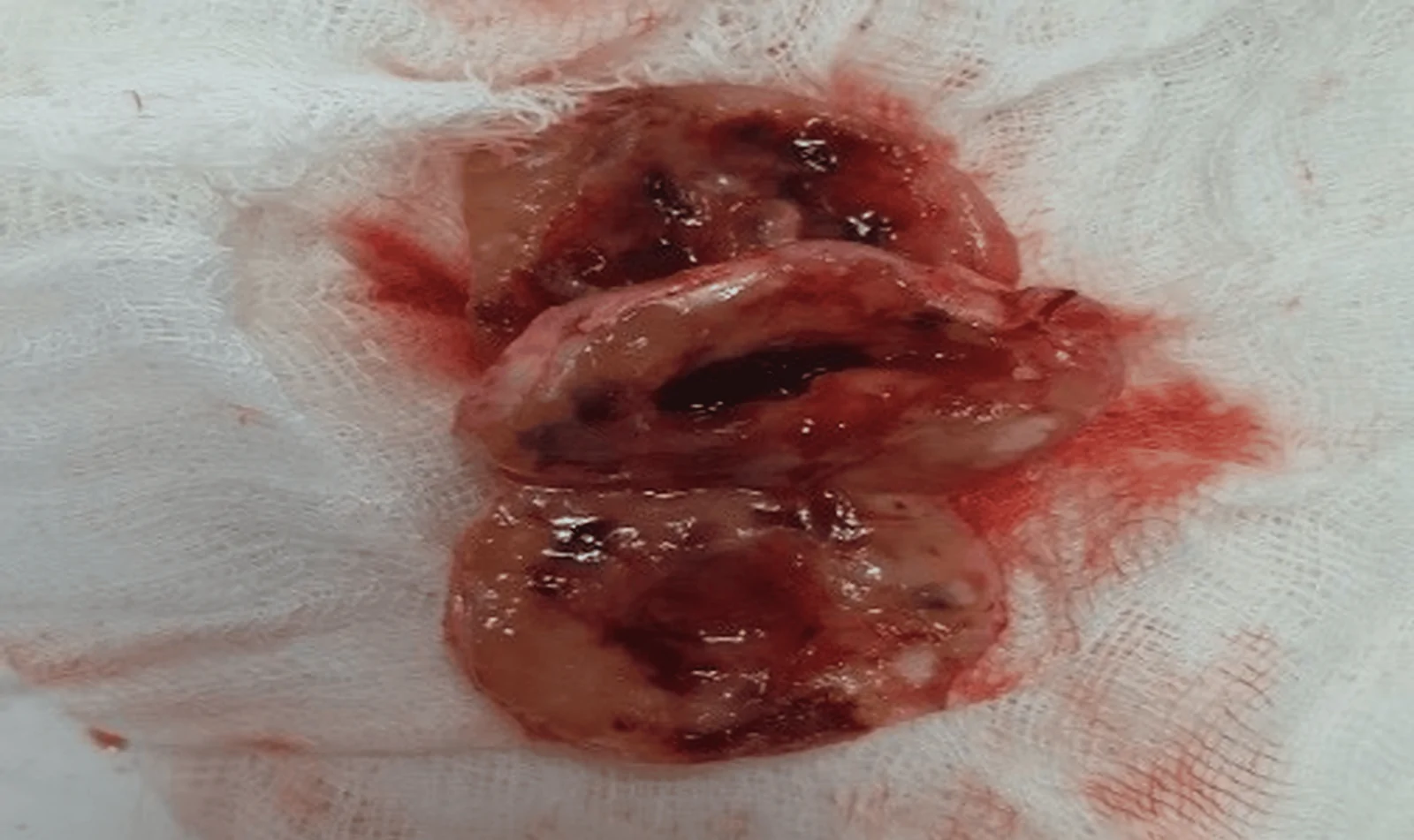
Cut section of the mass Cut section of the gross specimen showing solid and cystic areas

Microscopically, the tumor was surrounded by a thick collagenous capsule. It had moderate cellularity and rich vascularity. The tumor cells were stellate, lying in abundant extracellular myxoid material, and showed no atypia (Figure 3). On special staining, the tumor was positive for Alcian blue, showing mucin. On immunohistochemistry, the tumor cells were positive for vimentin (Figure 4) and negative for desmin and cytokeratin. From the HPE and immunohistochemistry, a final diagnosis of ovarian myxoma was made.

**Figure 3:**
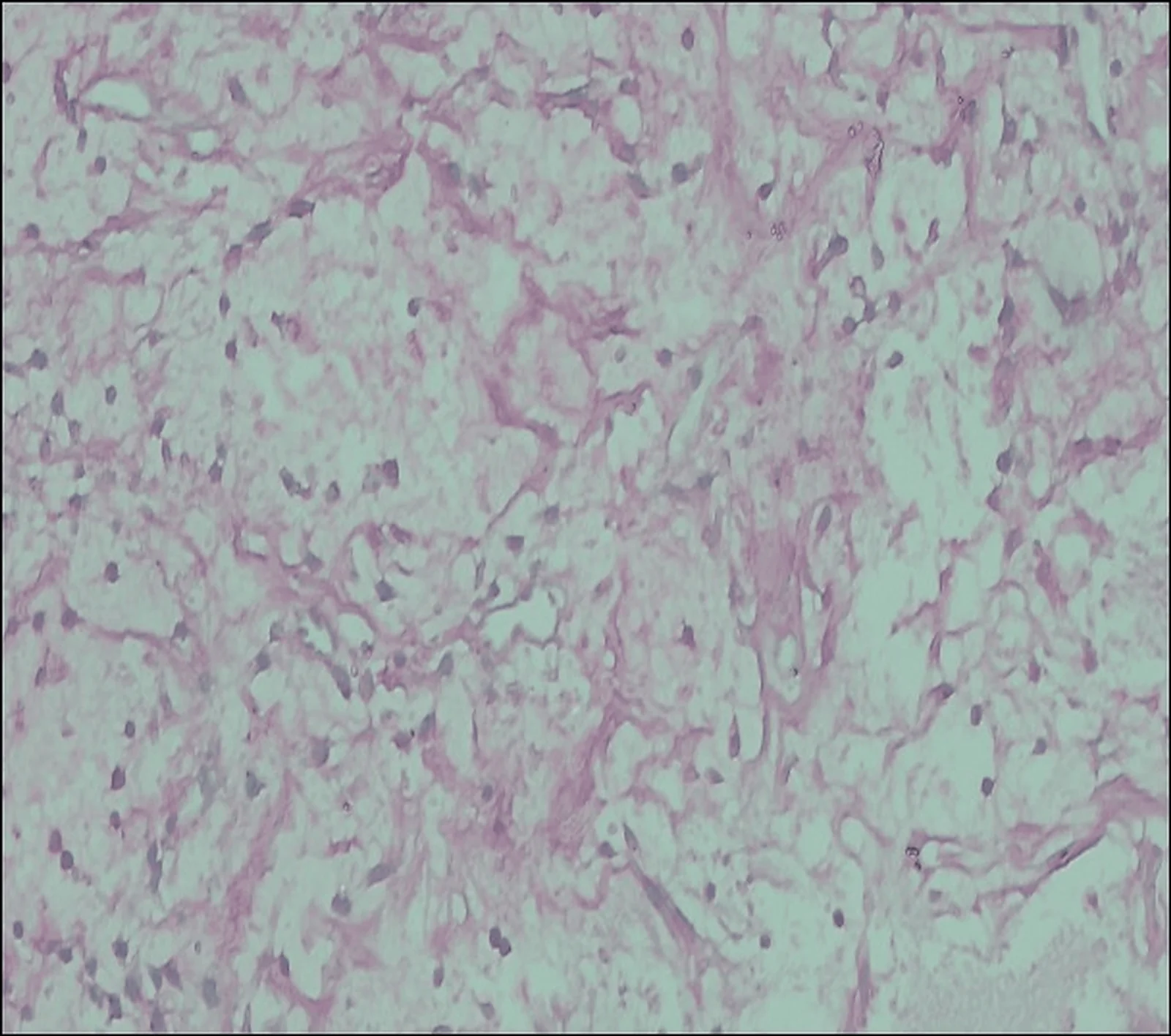
Hematoxylin and eosin stain of the tumor Microscopic view showing stellate cells lying in abundant myxoid stroma, without atypia

**Figure 4:**
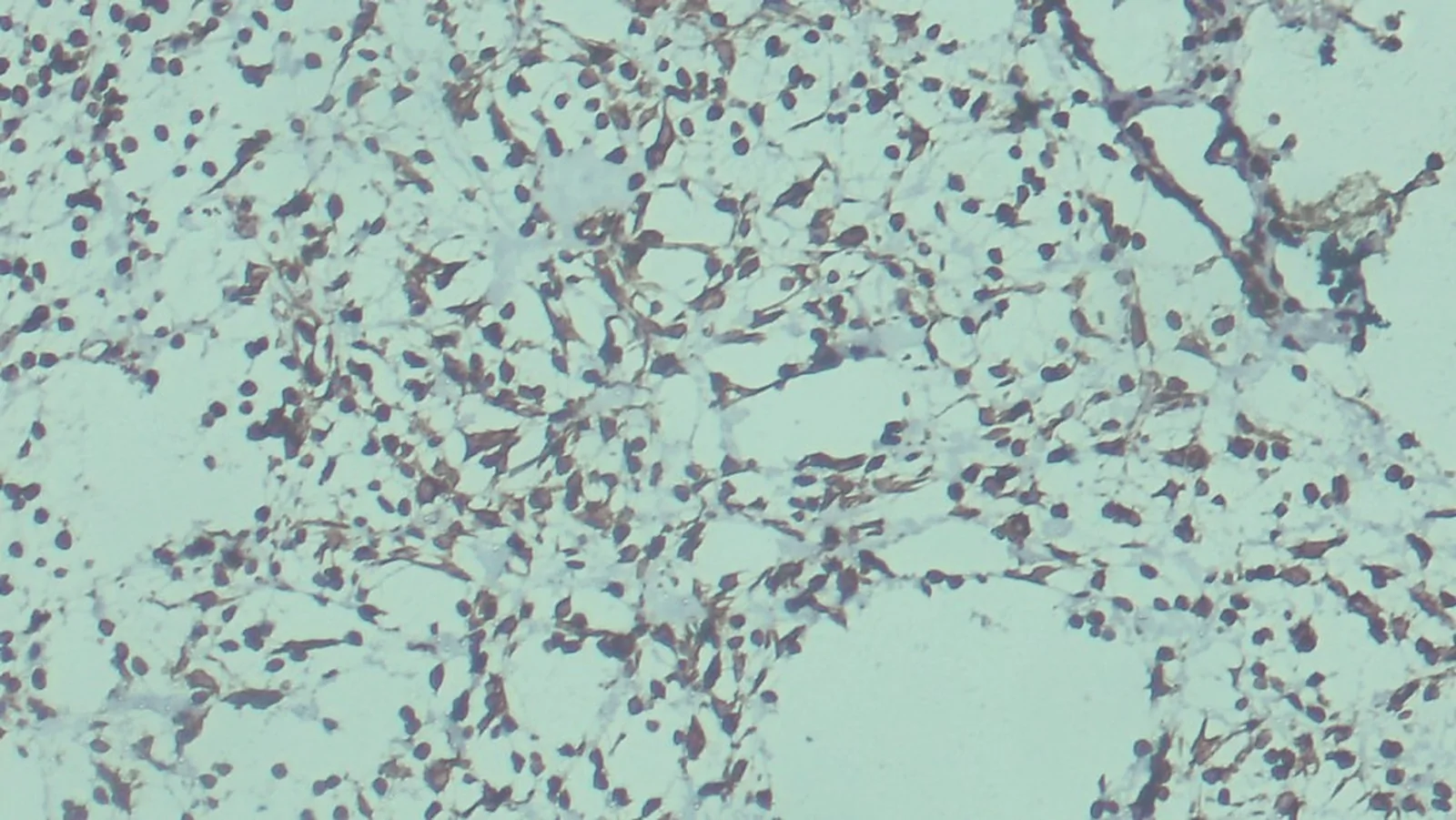
Vimentin immunohistochemistry of the ovarian myxoma Diffuse brown cytoplasmic vimentin positivity in the stellate and spindle-shaped tumor cells

Outcome

The postoperative course was uneventful, and she was discharged on postoperative day 5. Biopsies from the left ovary and the omentum showed no tumor. At one-year follow-up, there was no clinical or ultrasonographic evidence of recurrence.

## Discussion

Ovarian myxoma is a rare ovarian tumor with typical myxoid features. In 1991, Eichhorn and Scully did clinicopathologic and immunocytologic analysis of five cases of ovarian myxoma. They reported that patients commonly had an asymptomatic unilateral adnexal mass and the tumors looked solid and cystic in varying degrees [1]. Other reported cases also showed nonspecific clinical and imaging findings [2]. Rix et al. reported another case of ovarian myxoma in 1998, further emphasizing the rarity of this tumor [3]. Because the imaging and surgical findings are not specific, it can be mistaken for other ovarian masses, such as a mature cystic teratoma. This matters in young women, because a wrong diagnosis can lead to more surgery than needed, including removal of the whole ovary.

The diagnosis was difficult at several stages in this case. The mass caused no symptoms and was found incidentally during the evaluation of a missed abortion. Adnexal masses are not uncommon incidental findings during pregnancy, and many are asymptomatic and detected during routine ultrasonography [4]. Imaging showed a complex solid-cystic lesion with hyperechoic foci and posterior acoustic shadowing, so a teratoma was suspected. Intraoperative scrape cytology showed squamous cells, columnar cells, and mature adipose tissue, which also favored a benign teratoma.

The final diagnosis was made on histopathology. The tumor had a thick collagenous capsule, moderate cellularity, and rich vascularity. Its stellate cells lay in abundant myxoid material without atypia. This picture fits ovarian myxoma [1,2,5]. On immunohistochemistry, vimentin positivity supports mesenchymal origin, and negativity for desmin and cytokeratin argues against smooth muscle or epithelial differentiation. Costa et al. demonstrated vimentin expression in ovarian myxomas, with immunohistochemical findings supporting their mesenchymal nature [5,6]. Immunohistochemistry should therefore be interpreted together with the characteristic morphology rather than in isolation [1,5,6]. However, the immunohistochemical panel in this case was limited, and additional markers, including inhibin, calretinin, smooth muscle actin, CD34, and Ki-67, were not performed. Alcian blue staining was positive, although the corresponding figure was unavailable. Further immunohistochemical characterization could have provided additional support for the diagnosis and assessing relevant differential diagnoses.

The differential diagnosis includes massive ovarian edema, edematous fibroma, myxoid change in ovarian stromal tumors, and malignant myxoid tumors. Eichhorn and Scully stressed the need to separate ovarian myxoma from massive edema, edematous fibroma, and myxoid sarcomas [1]. Massive ovarian edema is characterized by marked stromal edema with preservation of ovarian structures and may closely mimic an ovarian neoplasm. It should therefore be considered in the differential diagnosis of a solid or complex ovarian mass [7]. Rywlin specifically discussed the differential diagnosis between ovarian myxoma and massive ovarian edema and highlighted the usefulness of Alcian blue staining in this distinction [8]. Costa et al. suggested that ovarian myxoma may belong to the thecoma-fibroma group of ovarian stromal tumors, based on overlapping morphology and immunohistochemistry [6]. More recently, Roth et al. reported four ovarian myxomas, three of which were associated with other ovarian stromal tumors, supporting the possibility that some ovarian myxomas may arise through myxoid transformation of a pre-existing stromal tumor [9]. More recently, Meng et al. reported a case of ovarian myxoma associated with a sclerosing stromal tumor, further highlighting the possible relationship between ovarian myxoma and other ovarian stromal neoplasms [10]; the exact origin of the tumor is still not clear.

Malignant myxoid tumors should also be considered in the differential diagnosis. Myxoid leiomyosarcoma of the ovary is extremely rare but may present as an ovarian mass and requires distinction from benign myxoid lesions. Histological assessment for cytological atypia, mitotic activity, and other malignant features, together with appropriate smooth-muscle immunohistochemical markers, is important in establishing the diagnosis [11]. Primary ovarian myxoid liposarcoma is another rare malignant differential and is characterized by a delicate branching or “chicken-wire” vascular pattern and lipoblasts; demonstration of a CHOP/DDIT3 rearrangement may support the diagnosis [12]. In the present case, the absence of atypia, necrosis, and mitotic activity, together with the immunohistochemical profile, supported a benign ovarian myxoma.

In 2004, Kim et al. reported an antenatal case of ovarian myxoma in a 25-year-old woman whose ovarian mass was found by chance during antenatal evaluation [13]. That tumor was also mostly solid and made of stellate cells in abundant myxoid material, and it was reported as having uncertain malignant potential because of its high cellularity [13]. Our case differs in its clinical setting and in how the diagnosis unfolded, especially due to close resemblance to a mature cystic teratoma on imaging, at gross examination, and on scrape cytology.

Ovarian myxoma is generally considered benign, and long-term follow-up in two cases has been described [14]. However, Bedir et al. noted that its behavior is uncertain and recurrence may occur, and they advised complete excision of the tumor together with the adnexal structures [15]. Sterkers et al. subsequently discussed ovarian-sparing surgery in a young woman, highlighting the importance of fertility preservation in selected cases [16]. In our patient, cystectomy was done instead, which preserved ovarian tissue and is important for fertility preservation in a young woman.

## Conclusions

Ovarian myxoma is a rare benign ovarian mesenchymal tumor that can present as an incidental, asymptomatic adnexal mass and may closely mimic a mature cystic teratoma. In the present case, the lesion was discovered incidentally during evaluation of a missed abortion. The combination of a complex solid-cystic appearance with hyperechoic foci on imaging, benign-appearing intraoperative scrape cytology, and the absence of characteristic gross features made the diagnosis particularly challenging. HPE supported by immunohistochemistry helped establish the final diagnosis.

This case emphasizes that ovarian myxoma should be considered in the differential diagnosis of an ovarian mass when the clinical, radiological, and intraoperative findings are not entirely concordant. Intraoperative cytology may not reliably distinguish ovarian myxoma from other benign ovarian lesions. HPE therefore remains essential for definitive diagnosis, with immunohistochemistry providing additional diagnostic support. In a young patient, fertility-preserving surgery may be considered when complete excision is feasible and the clinical circumstances permit. The absence of recurrence at one-year follow-up in our patient is reassuring, although continued clinical follow-up remains appropriate. Overall, this case highlights the diagnostic challenge posed by this uncommon tumor and the importance of correlating imaging, intraoperative findings, morphology, and immunohistochemistry to reach the correct diagnosis.
